# Taking the lead: NLR immune receptor N‐terminal domains execute plant immune responses

**DOI:** 10.1111/nph.19170

**Published:** 2023-07-31

**Authors:** Khong‐Sam Chia, Philip Carella

**Affiliations:** ^1^ Cell and Developmental Biology John Innes Centre Colney Lane Norwich NR4 7UH UK

**Keywords:** cell death, coiled coil, evolution, immunity, receptors, Toll/interleukin‐1 receptor (TIR)

## Abstract

Nucleotide‐binding domain and leucine‐rich repeat (NLR) proteins are important intracellular immune receptors that activate robust plant immune responses upon detecting pathogens. Canonical NLRs consist of a conserved tripartite architecture that includes a central regulatory nucleotide‐binding domain, C‐terminal leucine‐rich repeats, and variable N‐terminal domains that directly participate in immune execution. In flowering plants, the vast majority of NLR N‐terminal domains belong to the coiled‐coil, Resistance to Powdery Mildew 8, or Toll/interleukin‐1 receptor subfamilies, with recent structural and biochemical studies providing detailed mechanistic insights into their functions. In this insight review, we focus on the immune‐related biochemistries of known plant NLR N‐terminal domains and discuss the evolutionary diversity of atypical NLR domains in nonflowering plants. We further contrast these observations against the known diversity of NLR‐related receptors from microbes to metazoans across the tree of life.

**Table:** 

	Contents	
	Summary	496
I.	Introduction	496
II.	NLR N‐terminal domains orchestrate plant immune responses	497
III.	On the origin and evolutionary diversity of plant NLR N‐terminal domains	498
IV.	An emerging diversity of NLR‐related immune processes across the tree of life	499
V.	Future perspectives and remaining questions	499
	Acknowledgements	500
	References	500

## I. Introduction

Nucleotide‐binding domain and leucine‐rich repeat proteins (NLR) are an essential component of the plant immune system. Acting as intracellular immune receptors, these proteins monitor host cells for perturbations caused by pathogen virulence effectors and subsequently activate robust immune responses to suppress pathogen ingress (Jones *et al*., 2016). In general, NLRs are composed of a canonical tripartite architecture, consisting of a variable N‐terminal domain, a central conserved NB‐ARC domain (named after Nucleotide‐Binding adaptor shared with APAF‐1, plant resistance proteins, and CED‐4), and superstructure forming C‐terminal leucine‐rich repeats (LRR). The biochemical activities of each NLR subdomain are well‐studied in angiosperm model systems and crops (reviewed in detail in Maruta *et al*., 2022; J. Wang *et al*., 2023). Briefly, the C‐terminal LRR region is associated with pathogen effector recognition (direct or indirect) as well as receptor autoinhibition to prevent spurious immune activation. Upon effector recognition, NLRs undergo a conformational change to facilitate an adenosine diphosphate (ADP)‐to‐adenosine triphosphate (ATP) exchange within the P‐loop region of the regulatory NB‐ARC domain that functions as an on–off switch. Activated NLRs then form higher order oligomer complexes that enable N‐terminal domain multimers to perform immune‐related biochemical functions that the monomer is incapable of. In this insight review, we focus on the variable N‐terminal domains of plant NLRs and discuss their known biochemical activities, evolutionary and functional diversity, and emerging commonalities with noncanonical NLR‐like proteins from across the tree of life.

## II. NLR N‐terminal domains orchestrate plant immune responses

The NLRs of flowering plants are generally categorized into three major groups based on the identity of their N‐terminal domain, which includes the four helix‐bundle containing coiled‐coil (CC) and Resistance to Powdery Mildew 8 alongside (RPW8) Toll‐like/Interluekin1 receptor (TIRs). These are often viewed as ‘signalling’ or ‘executioner’ domains since they encode the direct biochemical activity required for immune execution. Consequently, the overexpression of CC, RPW8, or TIR domains is often sufficient to activate plant immune responses (Swiderski *et al*., 2009; Bernoux *et al*., 2011; Bentham *et al*., 2018) that include the accumulation of defense hormones, drastic transcriptional reprogramming, rapid release of reactive oxygen species, and hypersensitive response‐type cell death (Lolle *et al*., 2020; Dalio *et al*., 2021).

**Fig. 1:**
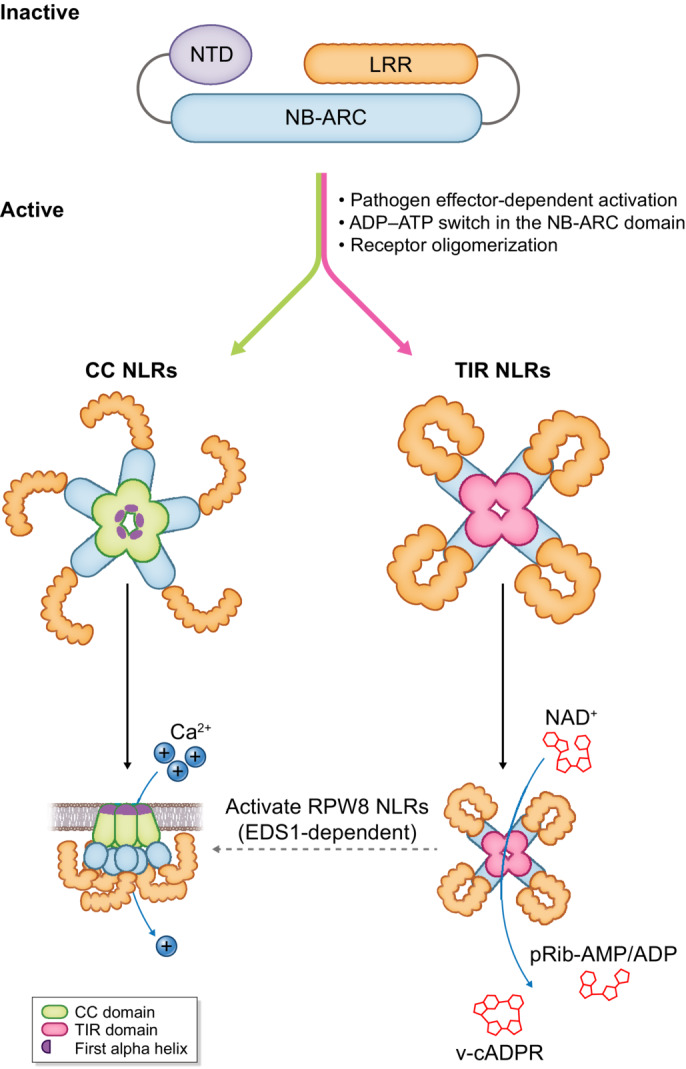
Activated nucleotide‐binding domain and leucine‐rich repeat (NLR) oligomers enable N‐terminal domain immune functions. In the absence of pathogens, plant NLRs remain inactive. This is maintained by tight interactions between the leucine‐rich repeat (LRR) and nucleotide‐binding adaptor shared with APAF‐1, plant resistance proteins, and CED‐4 (NB‐ARC) domains, which lock the receptor in an adenosine diphosphate (ADP)‐bound state to prevent auto‐activation. Upon pathogen effector‐mediated activation, a conformational change releases the NB‐ARC domain and allows for ADP‐adenosine triphosphate (ATP) exchange. This promotes NLR oligomerization, with coiled‐coil (CC) NLRs forming a pentameric complex that binds to the plasma membrane. The first alpha helix of this CC complex forms a channel‐like funnel structure that triggers calcium ion influx and ultimately leads to hypersensitive‐response (HR) cell death. In comparison, tetrameric Toll/interleukin‐1 receptor (TIR)‐type NLR complexes form a holoenzyme complex that utilizes nicotinamide adenine dinucleotide (NAD) as a substrate to generate immune‐related small molecules that trigger downstream immune responses through Resistance to Powdery Mildew 8 (RPW8)‐type NLRs in an ENHANCED DISEASE SUSCEPTIBILITY1 (EDS1)‐dependent manner. Helper RPW8‐NLRs are predicted (dashed arrow) to oligomerize and function in similar manner as CC‐NLRs, though additional structural and biochemical studies are needed to confirm the existence of RPW8‐NLR ‘resistosomes’ and understand how the EDS1 pathway integrates into this framework.

Emerging structural and biochemical studies have revealed new mechanistic insights into NLR functionality in flowering plants. In general, pathogen‐induced activation of CC and TIR‐type NLRs promotes receptor oligomerization leading to multimeric N‐terminal domain complexes capable of executing immune‐relevant biochemistry (Fig. 1). This can be achieved directly within a single NLR (singleton) or can be distributed between pairs/networks of sensor NLRs (pathogen detector) that activate helper NLRs (immune executors). Cryo‐EM studies of the pentameric CC‐NLR ‘resistosomes’ of *Arabidopsis* (ZAR1) and wheat (Sr35) singleton NLRs demonstrate that the first alpha helix of each CC domain forms a channel‐like funnel structure that likely targets the plasma membrane and alters calcium ion flux (Wang *et al*., 2019; Förderer *et al*., 2022). In further support of this model, helper CC‐NLRs also form homo‐oligomeric complexes upon sensor activation and accumulate at membrane‐bound puncta (Adachi *et al*., 2019; Bi *et al*., 2021; Ahn *et al*., 2023; Contreras *et al*., 2023). Moreover, singleton/helper CC‐NLRs typically encode N‐terminal ‘MADA’ or ‘MADA‐like’ motifs within the first alpha helix that are essential for cell death induction (Adachi *et al*., 2019). An equivalent mechanistic model is proposed for RPW8‐type helper NLRs that similarly associate with the plasma membrane, alter calcium ion permeability, depend on N‐terminal alpha helix  motifs, and oligomerize upon immune activation (Jacob *et al*., 2021; Saile *et al*., 2021; Feehan *et al*., 2023).

High‐resolution cryo‐EM structures of effector‐activated TIR‐NLR receptors from *Arabidopsis* (RPP1) and *Nicotiana* (ROQ1) provided further biochemical insights on TIR‐mediated plant immunity. Here, effector‐dependent activation leads to a tetrameric receptor configuration whereby asymmetrically aligned TIR domain homodimers reconstitute a NAD (nicotinamide adenine dinucleotide) hydrolyzing holoenzyme complex (Ma *et al*., 2020; Martin *et al*., 2020). In addition, TIR domains exhibit 2′,3′‐cAMP/cGMP synthetase activity through the direct binding and hydrolysis of dsRNA/dsDNA (Yu *et al*., 2022). The bifunctional biochemical activities of TIRs leads to the accumulation of an array of immunogenic small molecules (pRib‐AMP/ADP, diADPR/ADPr‐ATP, 2′‐cADPR, 3′‐cADPR, or 2′,3′‐cAMP/cGMP), of which pRib‐AMP/ADP or diADPR/ADPr‐ATP directly bind and stimulate ENHANCED DISEASE RESISTANCE1 (EDS1)‐based regulatory complexes (Wan *et al*., 2019; Huang *et al*., 2022; Jia *et al*., 2022; Bayless *et al*., 2023). Activated EDS1 complexes (containing either PAD4 or SAG101) then trigger distinct helper RPW8‐NLRs (ADR1 or NRG1), leading to receptor oligomerization and downstream RPW8‐dependent biochemical activities (described above) that promote cell death (Fig. 1).

## III. On the origin and evolutionary diversity of plant NLR N‐terminal domains

**Fig. 2:**
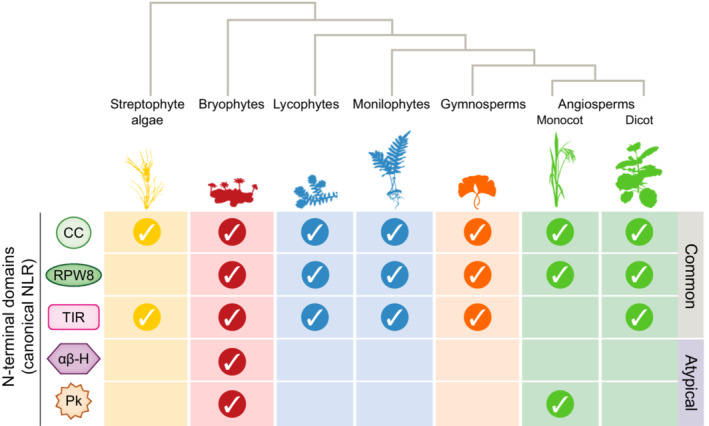
The plant kingdom harbors common and atypical nucleotide‐binding domain and leucine‐rich repeat (NLR) N‐terminal domains. A simplified phylogeny of land plants (not to scale) that includes streptophyte algae (sister to land plants), followed by bryophytes (nonvascular, nonseed), lycophytes and monilophytes (vascular, nonseed), gymnosperms (vascular, seed, nonflowering), and angiosperms (flowering seed plants, generally subdivided into monocots and dicots). The overall presence/absence of canonical NLR receptor architectures harboring N‐terminal domains of common (CC, coiled‐coil; RPW8, Resistance to Powdery Mildew 8; TIR, Toll/interleukin‐1 receptor) or atypical αβ‐hydrolases (αβ‐H) and protein kinase (Pk) domains are indicated per lineage/group.

Experimental research on plant NLRs has focused solely on flowering plant (angiosperm) model systems and crops, with all functionally validated receptors belonging to this lineage (Kourelis *et al*., 2021). However, the plant kingdom harbors a much wider diversity of lineages that includes streptophyte algae (sister to all land plants), nonvascular/nonseed bryophytes (liverworts, hornworts, and mosses), vascular/nonseed lycophytes and monilophytes (clubmosses and ferns), and vascular/seed‐bearing gymnosperms (conifers, cycads, ginkgo, and gnetophytes). The increasing availability of high‐quality plant and algal genomes has facilitated the identification and bioinformatic exploration of NLRs across the plant kingdom, enabling a greater macroevolutionary understanding of their history and diversity (Gao *et al*., 2018; Andolfo *et al*., 2019; Van Ghelder *et al*., 2019; Chia *et al*., 2022). Although limited in breadth when compared against angiosperms, these surveys demonstrate the presence of both common (CC, RPW8, and TIR) and atypical (αβ‐hydrolases and protein kinases) N‐terminal NLR domains in nonflowering plants (overview in Fig. 2).

The N‐terminal domain subtypes of angiosperms are widely distributed amongst the plant kingdom, which suggests their evolutionary conservation. These domain configurations likely originated during (or prior to) the emergence of green algae, as streptophytes such as *Chara braunii*, *Chlorokybus atmophyticus*, and *Klebsormidium nitens* harbor NLR‐like receptors (truncated or harboring alternative C‐terminal repeats) containing TIRs or CC/RPW8‐like 4 helix bundles (Gao *et al*., 2018; Andolfo *et al*., 2019; Chia *et al*., 2022), while the chlorophyte *Chromochloris zofingiensis* (unicellular green alga) encodes TIR‐NLRs (Andolfo *et al*., 2019). Emerging evidence supports the idea that the widely distributed N‐terminal CCs, RPW8s, and TIRs are functionally conserved across the green lineage, as domains from streptophyte algae and nonflowering plants activate cell death when transferred to the angiosperm *Nicotiana benthamiana* (Chia *et al*., 2022). Despite their high prevalence and functional conservation, these N‐terminal domains also display signatures of diversification between lineages. For example, the sequence divergent CC domains of nonflowering plants encode a distinct N‐terminal ‘MAEPL’ motif in the first alpha helix that is responsible for cell death activity in a manner comparable to the MADA motif of angiosperm CC‐NLRs (Chia *et al*., 2022). This suggests that the diverse CC domains of nonflowering plants form ion permeable pores similar to flowering plants. In addition, monocots (angiosperm subgroup) and liverworts have both lost the canonical TIR‐NLR architecture and instead encode TIR‐NB‐ARC‐TPR (TNP) NLR‐like receptors that may play a role in defense (Baggs *et al*., 2020; Johanndrees *et al*., 2022).

Excitingly, the macroevolutionary exploration of plant NLRs has identified unique NLR configurations that include N‐terminal αβ‐hydrolase or protein kinase domains rather than the more common CC/RPW8 or TIR domains (Gao *et al*., 2018; Andolfo *et al*., 2019; Chia *et al*., 2022). These atypical domains first appear in the nonvascular/nonseed bryophytes, which represent the earliest lineage to diverge from the last common ancestor of land plants. The N‐terminal αβ‐hydrolase is most prominently abundant in liverworts such as *Marchantia polymorpha* and in *Sphagnum* and *Ceratodon* mosses. While we know little about its precise biochemical function(s), its broad classification as an αβ‐hydrolase similar to lipase‐like proteins hints toward potential involvement in enzymatic hydrolysis and/or binding to lipids or lipid‐like molecules. Consequently, the biochemical outputs of these hydrolases may share functional similarity with TIRs (hydrolysis), CC/RPW8s (membrane/lipid‐binding), or even EDS1 (lipase‐like protein regulator of TIR‐NLR signaling); however, an entirely novel immune‐related biochemistry may also be awaiting discovery. Protein kinase‐type NLRs are prominently expanded in mosses but are also present in monocots. The biochemical activity of the protein kinase N‐terminal domain is conceptually straightforward (phosphorylation of target proteins), though we still require experimental evidence to support direct involvement in immune execution. At present, the best characterized kinase‐NLR is the Tsn1 receptor from wheat. Rather than contributing to immunity, Tsn1 is a susceptibility factor that facilitates disease upon the recognition of ToxA effectors from necrotrophic fungal pathogens. It remains unclear whether the N‐terminal kinase domain is directly responsible for this outcome; however, mutations in the kinase domain can abrogate Tsn1 function much like mutations in the NB‐ARC or LRRs (Faris *et al*., 2010). Monocots also harbor NLRs with N‐terminal BED‐type zinc finger domains (Marchal *et al*., 2018), though their role in immune execution and/or potential to bait pathogen effectors as integrated decoys remains to be clarified (Sanchez‐Martin & Keller, 2021). Collectively, these studies hint toward an extended array of NLR N‐terminal domain functions across the full spectrum of plant diversity. Whether these atypical domains directly participate in novel immune biochemistry, are co‐opted by pathogen effectors/toxins to promote disease, or were recruited for biological functions outside the realm of plant–microbe interactions remains to be discovered.

## IV. An emerging diversity of NLR‐related immune processes across the tree of life

Nucleotide‐binding domain and leucine‐rich repeat‐type receptors are an essential component of organismal immunity across the tree of life. Much like plants, receptors from bacteria, fungi, and metazoans consist of a tripartite architecture where the N‐terminal domain encodes immune‐related biochemical activities. Cryo‐EM studies of the bacterial SeAvs3 and EcAvs4 receptor complexes demonstrate receptor tetramerization upon phage detection that brings N‐terminal nuclease ‘effector’ domains together to enable DNA binding and hydrolysis (Gao *et al*., 2022). Computational and functional explorations of bacterial NLR‐related proteins reveals a wide diversity of N‐terminal domains that includes TIRs, hydrolases, caspases, proteases, transmembrane domains, and various endonucleases among other domain subtypes (Kibby *et al*., 2023). This range of diversity is also observed in fungi, where NLRs similarly harbor TIRs, CC‐like domains (MLKL, HeLo, Goodbye), kinases, αβ‐hydrolases, and patatin phospholipases (Wojciechowski *et al*., 2022). Several of these domains contribute to programmed cell death (Daskalov *et al*., 2016; Uehling *et al*., 2017), including the unique N‐terminal patatin phospholipase domain of the PLP‐1 receptor from *Neurospora crassa* (Heller *et al*., 2018). In comparison, metazoans (animals) encode NLRs with one of four major N‐terminal domain subgroups: CARD (caspase activation and recruitment domains), PYD (pyrin domain), BIR (baculovirus inhibitor of apoptosis protein repeat), or an acidic transactivating domain (Ting *et al*., 2008). Much like plants and bacteria, activated metazoan NLRs form large ‘inflammasome’ complexes that bring N‐terminal domains into proximity (Shen *et al*., 2019). However, metazoan NLR N‐terminal domains serve as platforms to recruit and/or activate downstream signaling proteins. For example, CARD–CARD interactions within the NLRC4 inflammasome facilitates the recruitment, dimerization, auto‐proteolytic processing, and activation of the downstream signaling regulator caspase‐1 (Zhang *et al*., 2015; Li *et al*., 2018). Macroevolutionary surveys of animal NLRs suggests their emergence in invertebrate sponges such as *Amphimedon queenslandica*, with divergent metazoan NLRs carrying prototypical N‐terminal CARD domains (and similar ‘DEATH’‐folds) amongst a highly variable receptor pool that is consistent with predicted roles in immunity (Yuen *et al*., 2014; Zhu *et al*., 2022).

## V. Future perspectives and remaining questions

The fact that NLRs and their diverse N‐terminal domains are commonly observed across the tree of life suggests their fundamental involvement in host immunity. In some instances, these broadly distributed domains are functionally conserved, like the TIR domains of plant, bacterial, and animal immune receptors that exhibit cross‐reactivity when transferred between kingdoms (Wan *et al*., 2019; Ofir *et al*., 2021; Bayless *et al*., 2023). By contrast, other N‐terminal domains may have emerged by convergent evolution to target similar processes, as is likely for the diversity of four helix bundle CC‐like domains that similarly target host membranes (Daskalov *et al*., 2016; Jacob *et al*., 2021; Chia *et al*., 2022; Förderer *et al*., 2022). Recent protein structural studies underscore the importance of the canonical NLR architecture in providing a platform for the multimerization of N‐terminal domains. We therefore hypothesize that receptor oligomerization is similarly required for the activity of atypical NLR N‐terminal domains. While this suggests an exciting potential for novel immune biochemistry, we cannot rule out the possibility that these domains act as baits for pathogen effector detection or as scaffolds for downstream protein–protein interactions. It is also possible that the atypical NLRs of nonflowering plants perform functions in plant processes other than immunity (discussed in Box 1). Lastly, an understanding of how pathogenic microbes interfere with N‐terminal domain biochemistry will provide further insight into NLR‐mediated immunity. As an example, emerging research demonstrates that the bacterial phytopathogen *Pseudomonas syringae* utilizes virulence effectors that disrupt TIR‐type (hopAM1, hopBY1) and RPW8‐type (AvrPtoB) NLR immune receptors (Eastman *et al*., 2022; Hulin *et al*., 2023; M.‐Y. Wang *et al*., 2023). We therefore propose that an extended set of model hosts and pathogens is needed to fully leverage the evolutionary diversity of NLR‐mediated immune responses in plants.

Box 1 **Roles for plant NLRs beyond immunity**

Nucleotide‐binding domain and leucine‐rich repeat receptors are implicated in plant processes other than immunity. For example, the TIR‐NLR protein CHS1 is required for balancing plant responses to cold temperatures in *Arabidopsis*, as *chs1‐1* mutants display a detrimental cold sensitivity dependent on TIR‐signaling machinery such as EDS1 and PAD4 (Zbierzak *et al*., 2013). Additional studies have identified distinct TIR‐NLRs impacting abiotic stress responses, including the ACQOS (ACQUIRED OSMOTOLERANCE) receptor in osmotic tolerance (Ariga *et al*., 2017) and SNC1 (SUPRESSOR OF NPR1 CONSTITUTIVE) in heat tolerance (Zhu *et al*., 2010). Moreover, the RPW8‐type helper NLR ADR1 confers enhanced tolerance to drought when overexpressed in *Arabidopsis*, though this compromises salt and heat stress responses (Chini *et al*., 2004). Collectively these studies demonstrate key roles for NLRs and NLR‐mediated immune regulators in abiotic stress pathways. In further support of this idea, comparative genomic analyses revealed a synchronized co‐elimination of the EDS1/PAD4 immune pathway (inclusive of TIR‐NLRs) with genes implicated in drought stress (Baggs *et al*., 2020). Together these findings highlight the intersection of biotic and abiotic stress responses in plants, suggesting that the role of NLRs may be more substantial and multifaceted than previously recognized. Considered alongside the well‐established role of NLRs as genetic incompatibility loci impacting hybridization (reviewed in Calvo‐Baltanas *et al*., 2021), it is therefore critical to consider the possibility that the atypical NLRs of nonflowering plants are involved in processes other than immunity.

## Competing interests

None declared.
